# The impact of attentional demand on the motor interference effect induced by dangerous stimuli

**DOI:** 10.7717/peerj.21820

**Published:** 2026-10-13

**Authors:** Shuyan Fan, Yang Gao, Congqian Xue, Wenhan Zhang, Kuiyuan Qin, Peng Liu

**Affiliations:** School of Public Administration/School of Emergency Management, Northwest University, Xi’an, Shaanxi, China

**Keywords:** Dangerous stimuli, Motor interference effect, Signal inhibition theory, Cognitive resource limited capacity theory, Attentional demand

## Abstract

The mechanisms by which attentional demand modulates the motor interference effect induced by dangerous stimuli remain debated. To test the competing predictions of the limited capacity theory and the signal suppression hypothesis, we manipulated attentional demand (low *vs*. high) and interfering stimulus type (high-arousal dangerous, medium-arousal dangerous, low-arousal neutral) within a Go/NoGo task combined with an additional singleton paradigm. Twenty-five healthy participants were recruited; after applying the *a priori* exclusion criterion, 24 were retained for analysis. Under low attentional demand, reaction times (RTs) did not differ across interfering stimulus types. Under high attentional demand, RTs were significantly longer for high-arousal dangerous stimuli than for low-arousal neutral stimuli and were marginally longer for medium-arousal dangerous stimuli than for low-arousal neutral stimuli. Error rates did not differ significantly across interfering stimulus types under either attentional-demand condition. The RT pattern was consistent with greater interference from more arousing dangerous stimuli under high attentional demand. These findings are consistent with the signal suppression hypothesis: when attentional resources are sufficient, task-irrelevant dangerous stimuli may be more effectively suppressed; when resources are taxed by a demanding primary task, suppression may be compromised, allowing dangerous stimuli greater access to task processing. However, attentional demand covaried with target stimulus type, so the observed interaction cannot be attributed to attentional demand alone; the equiprobable Go/NoGo design likewise precludes conclusions about motor inhibitory control. Boundary conditions and alternative interpretations are discussed.

## Introduction

Effectively avoiding dangerous objects is a crucial survival skill. Dangerous objects are stimuli that may pose potential harm, often eliciting heightened vigilance, and the motor interference effect they induce directly influences avoidance efficiency. Operationally, the motor interference effect is defined as the significant prolongation of reaction times (RTs) and/or increase in errors on Go trials when task-irrelevant dangerous background stimuli are present, relative to neutral background stimuli (Anelli, Borghi & Nicoletti, 2012; Anelli et al., 2013a; Liu et al., 2017). This behavioral interference is thought to reflect automatic attentional capture by danger-related information, which competes with ongoing task-relevant processing for limited cognitive resources. Prior research has identified the motor interference effect at the behavioral level (Anelli, Borghi & Nicoletti, 2012; Anelli et al., 2013a, 2013b) and has linked it physiologically to motor inhibition processing (Mustile et al., 2021; Wang & Liu, 2023) and danger evaluation (Cao, Cao & Liu, 2020). In the present framework, “dangerous” refers specifically to the capacity of a stimulus to signal potential physical harm and to activate defensive motivational systems, a construct distinct from general negative valence (or the perception of imminent personal threat, for conceptual delineation) (Anelli et al., 2013b; Morrison et al., 2007). Furthermore, the magnitude of this effect is modulated by the arousal level of the interfering stimulus: highly arousing stimuli tend to amplify motor interference, an amplification attributed to the greater cognitive resources required for comprehensive danger evaluation (Cao & Liu, 2021).

Given that dangerous stimuli automatically engage attention and motor systems, a critical question arises: how does the availability of cognitive resources modulate this automatic processing? Examining this question can illuminate how individuals avoid dangerous objects under the high cognitive demands characteristic of real-world situations. In daily life, contact with dangerous objects often occurs during states of inattention (Anelli et al., 2013a). Experimental paradigms accordingly superimpose target stimuli onto dangerous backgrounds to simulate such accidental contact: action signals in the foreground demand active processing, while dangerous objects occupy the background as task-irrelevant distractors. For instance, when green or red circles are superimposed on dangerous or safe objects, participants perform a Go/NoGo task on circle color while the background objects serve as interfering stimuli (Yiend, 2010). Critically, when such interfering stimuli receive insufficient cognitive resources, danger processing during action execution is impaired, potentially weakening the motor interference effect (Ogawa, Siiskonen & Hartikainen, 2012) and increasing the risk of unintentional contact.

Existing theories, however, make conflicting predictions about how cognitive resources influence the motor interference effect. The limited capacity theory of cognitive resources posits that individuals possess a finite resource pool, with both target and interfering stimulus processing drawing from this pool (Baror & Bar, 2022; Lavie, Ro & Russell, 2003; Lavie & Tsal, 1994). On this account, when the primary task demands substantial resources, fewer resources remain for processing interfering stimuli, which should weaken the motor interference effect. Most prior Go/NoGo studies of motor interference, however, have used relatively simple primary tasks—such as identifying dot color or discriminating the letters “W” and “M”, leaving sufficient residual resources for interference processing (Kahneman, 1973). Critically, such simple tasks fail to replicate the attentional demands of high-load real-world situations; the influence of interfering stimuli under high attentional demand therefore requires direct empirical examination (Cao & Liu, 2021; Liu et al., 2017).

The signal suppression hypothesis offers a contrasting prediction. According to this view, dangerous stimuli automatically capture attention, but top-down attentional control mechanisms actively suppress task-irrelevant salient stimuli (Gaspelin, Leonard & Luck, 2015; Sawaki & Luck, 2010). Supporting evidence comes from studies which found that under low attentional demand, threatening faces elicited larger early posterior negativity (EPN) and late positive potential (LPP) amplitudes than under high attentional demand, suggesting that top-down suppression, more effectively deployed when resources are ample, attenuates late-stage processing of dangerous distractors (Chen et al., 2023; Erthal et al., 2006; Hu, Ding & Qu, 2019). Extending this logic, we hypothesize that as primary-task cognitive demands increase, the resources available for suppressing interfering stimuli become insufficient, thereby potentiating the motor interference effect.

An important body of evidence, however, challenges the assumption that dangerous stimuli automatically capture attention irrespective of attentional demands (Pessoa et al., 2002). Mirabella, Grassi & Bernardis (2024), Mirabella et al. (2024) have demonstrated across multiple paradigms that emotional stimuli modulate behavioral performance only when their emotional dimension is task-relevant. For example, in Go/NoGo tasks where facial expressions (angry *vs*. happy) were either task-relevant (respond based on emotion) or task-irrelevant (respond based on gender), emotional expressions influenced RTs and error rates exclusively under task-relevant conditions (Mirabella, Grassi & Bernardis, 2024; Mirabella et al., 2024; Montalti & Mirabella, 2023, 2024). This task-relevance constraint appears robust: task-irrelevant emotional distractors consistently fail to affect performance, regardless of cognitive load (Montalti & Mirabella, 2024). These findings raise an important question for the present study: can task-irrelevant dangerous background stimuli nevertheless produce motor interference in the present paradigm? If so, this would suggest that the influence of task relevance may depend on stimulus and task characteristics, rather than demonstrating that dangerous stimuli necessarily bypass goal-dependent attentional selection. To test the competing predictions of the limited-capacity and signal-suppression accounts, the present study directly manipulated attentional demand within a motor-interference paradigm (Mirabella & Montalti, 2025).

Accordingly, the signal suppression hypothesis predicts a smaller motor interference effect under low than under high attentional demand, the opposite of what the limited capacity theory predicts. To test these competing predictions, we adopted the paradigm developed by Liu et al. (2017), incorporating the additional singleton paradigm (Sawaki & Luck, 2010) alongside the Go/NoGo task to independently manipulate attentional demand and interfering stimulus type.

Briefly, participants performed a Go/NoGo task in which prime stimuli (left or right hand images; here a prime serves as a response-preparation cue rather than a conventional perceptual or semantic prime) induced action preparation; subsequent target displays containing an additional singleton then required discrimination under low (line orientation) or high (letter identity) attentional demand. Interfering stimulus type was manipulated across three conditions, including high-arousal dangerous, medium-arousal dangerous, and neutral low-arousal, within a fully crossed 3 (interfering stimulus type) × 2 (attentional demand) × 2 (response type: Go/NoGo) within-subjects design, with reaction time and error rate as dependent variables.

Our hypotheses are as follows. According to the limited capacity theory, under low attentional demand the primary task consumes few resources, leaving sufficient residual capacity for processing interfering stimuli. This should produce a robust motor interference effect, significantly longer RTs on Go trials for high- and medium-arousal dangerous stimuli relative to neutral stimuli. Under high attentional demand, increased primary-attentional demands should constrain resources available for distractor processing, attenuating or eliminating the motor interference effect.

The signal suppression hypothesis generates the opposite predictions. Under low attentional demand, ample resources should support attentional suppression of task-irrelevant dangerous stimuli, attenuating or eliminating the motor interference effect. Under high attentional demand, depleted suppression resources should allow dangerous stimuli to escape suppression and undergo further processing, thereby enhancing motor interference. Critically, the hypothesized suppression operates at the level of attentional selection, preventing or reducing distractor processing, rather than at the level of motor response inhibition, which is typically indexed by successful withholding of prepotent responses in paradigms with unequal Go/NoGo probabilities (Wessel, 2018).

## Methods

### Participants

The minimum sample size required for the experiment was calculated using G*Power software, which indicated a requirement of 10 participants. Based on previous studies (Cao & Liu, 2021), the effect size was set at 0.4, with a significance level of 0.05 and statistical power of 0.95. To ensure the reliability of the findings, the study recruited 25 college students (12 males, 13 females), with a mean age of 22.8 years (*SD* = 2.4). During the data preprocessing phase, exclusion criteria were determined *a priori*: participants whose overall accuracy in the high attentional-demand condition fell more than three standard deviations below the group mean were to be excluded from analyses. The group mean accuracy under high attentional demand was 95.1% (*SD* = 5.1%), yielding an exclusion cutoff of 79.8%. One female participant (ID 23) was below this threshold (mean accuracy under high attentional demand was 79.5%). As a result, the remaining 24 participants (12 males, 12 females; mean age = 22.8 years, *SD* = 2.5) were retained for statistical analysis. All included participants met the following criteria: no history of mental illness or neurodevelopmental disorders. Prior to participation, all participants voluntarily signed an informed consent form, and the research protocol was approved by the Medical Ethics Committee of Northwest University (approval number 220128001).

### Materials and apparatus

Prime stimuli consisted of images of left or right hands in a grasping posture (visual angle: 13° × 11°), which were used to induce action preparation in a specific direction. Target stimuli comprised a background picture (visual angle: 13.4° × 10°) overlaid with a visual search array. Specifically, four gray geometric shapes (three circles and one diamond, or three diamonds and one circle; each shape subtended a visual angle of 2.5° × 2.5°) were presented equidistantly along an imaginary circle with a radius of 7.5°. Each shape contained either a white horizontal or vertical line, or the letters “E” or “F” (visual angle: 1.6°).

The background stimuli consisted of nine animal images selected from the International Affective Picture System (IAPS), Lang, Bradley & Cuthbert (2005). Among these, three images served as neutral low-arousal stimuli, three as medium-arousal dangerous stimuli, and three as high-arousal dangerous stimuli (Table 1). Table 2 presents the standardized IAPS valence and arousal ratings for each stimulus category. As expected, the dangerous images differed substantially from neutral images in both valence and arousal, while the key distinction between high- and medium-arousal threat categories was driven primarily by arousal rather than valence (see Table 2).

**Table 1:** Interfering stimuli and their codes in IAPS.

Conditions	Name
Dangerous high arousal	1050 (Snake1) 1120 (Snake2) 1300 (Dog)
Dangerous medium arousal	1220 (Spider) 1270 (Cockroach1) 1275 (Cockroach2)
Neutral low arousal	1333 (Parrot) 1450 (Waterbird) 1602 (Butterfly)

**Table 2:** Means and standard deviations of valence and arousal scores as a function of interfering stimulus type levels.

Conditions	Valence	Arousal
Dangerous high arousal	3.60 (0.17)	6.86 (0.07)
Dangerous medium arousal	3.48 (0.19)	5.12 (0.57)
Neutral low arousal	6.33 (0.20)	3.14 (0.30)

To verify the validity of the stimulus materials, an independent sample of 21 college students (who did not participate in the formal experiment) evaluated the animal images using a 5-point Likert scale on three dimensions: (1) stimulus danger (*i.e*., the tendency to elicit avoidance behavior), (2) visual complexity (*i.e*., the richness of visual elements), and (3) arousal ratings (ranging from calm/relaxed to excited/tense). One-way ANOVAs revealed a significant main effect of stimulus danger, *F*(2, 40) = 249.31, *p* < 0.001, *η^2^*_*p*_ = 0.93. Bonferroni-corrected *post hoc* tests indicated that high-arousal dangerous stimuli (*M* = 4.49, *SD* = 0.78) were rated as significantly more dangerous than medium-arousal dangerous stimuli (*M* = 2.60, *SD* = 0.75), which in turn were rated as significantly more dangerous than neutral stimuli (*M* = 1.18, *SD* = 0.42). The main effect of arousal was also significant, *F*(2, 40) = 29.51, *p* < 0.001, *η^2^*_*p*_ = 0.60, with ratings highest for high-arousal dangerous stimuli (*M* = 4.13, *SD* = 0.92), followed by medium-arousal (*M* = 3.03, *SD* = 1.12), and lowest for neutral (*M* = 2.21, *SD* = 1.03). In terms of visual complexity, ratings were similar across the three stimulus categories (high-arousal dangerous: *M* = 3.16, *SD* = 1.03; medium-arousal dangerous: *M* = 3.17, *SD* = 0.58; low-arousal neutral: *M* = 2.84, *SD* = 0.86). A one-way ANOVA confirmed no significant difference, *F*(2, 40) = 1.39, *p* = 0.26, *η^2^*_*p*_ = 0.07, indicating that visual complexity was well matched across categories. Furthermore, study-specific ratings were highly consistent with the IAPS normative values. Across the nine selected images, mean study-specific arousal ratings were strongly correlated with IAPS arousal values, *r*(7) = 0.98, *p* < 0.001. Mean study-specific danger ratings were strongly negatively correlated with IAPS valence values, *r*(7) = −0.97, *p* < 0.001, indicating that images rated as more dangerous in the study-specific ratings were also more negative according to IAPS norms.

The experiment was conducted on a Lenovo desktop computer connected to a 19-inch liquid crystal display (LCD) monitor with a refresh rate of 60 Hz. Stimulus presentation and response recording were controlled using E-Prime 2.0 software. Participants responded using a standard computer keyboard.

### Procedure

The experiment was conducted in a quiet, dimly lit laboratory room. Participants were seated 60 cm from the display screen, with their head position stabilized using an adjustable chin rest to ensure standardized visual presentation conditions.

Participants performed a two-stage decision on each trial. First, the prime image cued them to prepare a response with the corresponding hand (left index finger on the “Z” key and right index finger on the “M” key). They then located the additional singleton—the shape that differed from the other three—in the target display and discriminated the item within it: “E” *vs*. “F” in the high-demand condition or horizontal *vs*. vertical line orientation in the low-demand condition. Based on this discrimination, they either executed the prepared response (Go) or withheld it (NoGo).

The trial sequence is illustrated in Fig. 1. Each trial began with the presentation of a white fixation cross at the center of the screen for 300 ms, followed by a blank screen for 300 ms. A prime stimulus was then presented for 200 ms, followed by a 50-ms blank interval and the subsequent presentation of the target stimulus for up to 2,000 ms. Participants were instructed to respond within the 2,000-ms response window following target onset. The inter-trial interval varied randomly between 1,400 and 1,600 ms. To minimize eye fatigue and enhance stimulus visibility, all stimuli were presented against a black background.

**Figure 1:**
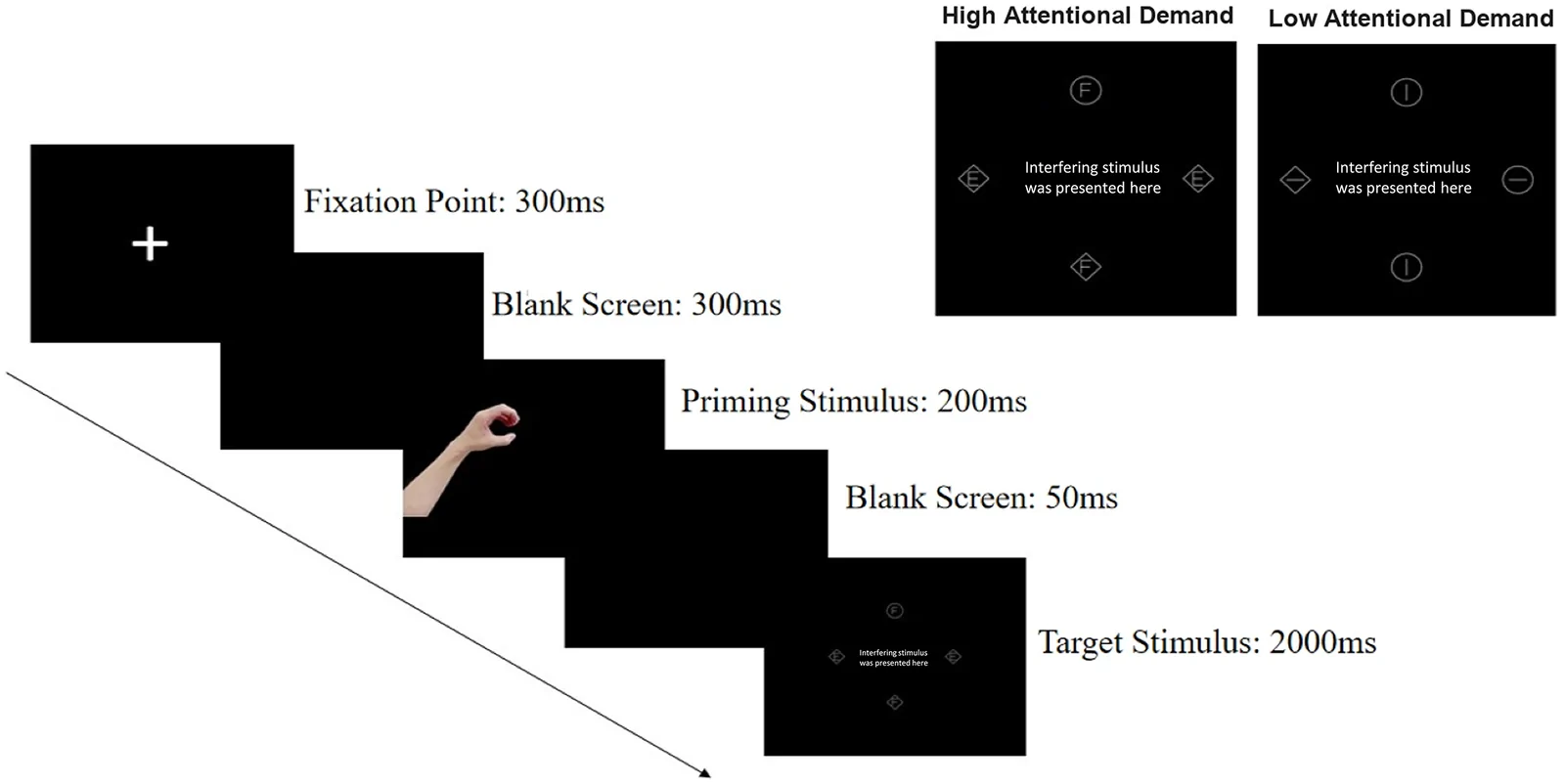
Example of the prime stimulus and experimental trial procedure.

The experiment employed a three-factor within-subjects design, manipulating interfering stimulus type (high-arousal dangerous, medium-arousal dangerous, neutral low-arousal), attentional demand (high, low), and response type (Go, NoGo). All participants completed two blocks corresponding to the high and low attentional-demand conditions. Each block consisted of 144 trials: three levels of interfering stimulus type × 2 response types (Go/NoGo) × 24 repetitions. With three images per stimulus category, each IAPS image appeared eight times per Go/NoGo condition per block, yielding 16 presentations per image per block (8 Go + 8 NoGo) and 32 presentations across the experiment. The diamond and circle additional singletons occurred equally often at each of the four possible spatial locations, and left- and right-hand prime stimuli were presented with equal probability. Prior to the formal experiment, participants completed nine practice trials per block to familiarize themselves with the task. To mitigate fatigue, a rest period of at least 2 min was provided after every 72 trials.

In the high attentional demand condition, participants were required to respond when the letter within the additional singleton was “E” (Go signal) and to withhold responses when it was “F” (NoGo signal). In the low attentional-demand condition, participants responded when the line segment within the additional singleton was horizontal (Go) and withheld responses when it was vertical (NoGo). Two counterbalancing schemes were applied across participants. Participants with odd-numbered IDs were assigned to one counterbalancing condition, whereas participants with even-numbered IDs were assigned to the other. First, response mapping was counterbalanced: participants with odd-numbered IDs (*N* = 12) used horizontal lines and the letter “E” as Go signals and vertical lines and “F” as NoGo signals, whereas participants with even-numbered IDs (*N* = 12) used the reverse mapping (vertical lines and “F” = Go; horizontal lines and “E” = NoGo). Second, block order was counterbalanced: odd-numbered participants completed the low attentional-demand block first (horizontal *vs*. vertical lines) followed by the high attentional-demand block (“E” *vs*. “F” letters), while even-numbered participants completed the high-demand block first.

It should be noted that the attentional-demand manipulation differed not only in task difficulty but also in target stimulus type: the high-demand condition required discrimination between letters (“E” *vs*. “F”), whereas the low-demand condition required discrimination between line orientations (horizontal *vs*. vertical). Previous visual-attention research indicates that discrimination of a basic orientation feature places relatively limited demands on focal attention compared with discrimination based on the configuration of component line segments (Cheal, Lyon & Hubbard, 1991). Letter identification has also been used as an attentionally demanding task relative to the detection of a basic orientation feature (Joseph, Chun & Nakayama, 1997). More broadly, perceptual load research has shown that task-relevant processing demand can be increased by requiring difficult stimulus identification rather than simple feature judgments (Lavie & Tsal, 1994). In the present task, “E” and “F” share most of their component strokes and differ primarily in the presence of one horizontal segment, whereas horizontal and vertical lines differ along a salient orientation dimension. The E/F discrimination was therefore expected to require finer-grained perceptual discrimination and greater attentional engagement. Nevertheless, because attentional demand and target stimulus type covaried, the present design cannot determine whether the observed effects were driven by attentional demand, stimulus-specific processing differences, or a combination of both. This limitation is considered further in the Discussion.

Go and NoGo trials were presented with equal probability (50% each). This equiprobable design differs from standard Go/NoGo paradigms in which Go trials typically predominate (70–80%) to establish a prepotent motor response (Wessel, 2018). The present paradigm was not designed as a canonical response-inhibition assay. Rather, it followed the motor-priming Go/NoGo paradigm developed by Cao & Liu (2021), Liu et al. (2017, 2023), in which a prime induces action preparation and the Go/NoGo signal serves as a response gate. The critical comparison concerns dangerous *vs*. neutral backgrounds on Go trials, when the prepared action is executed, rather than NoGo trials, which primarily index response withholding. The 50:50 ratio provided balanced trial counts across the fully crossed design (3 stimulus categories × 2 Go/NoGo × 2 attentional-demand levels) without confounding stimulus type with Go/NoGo probability. Prior ERP studies using this paradigm have associated the motor interference effect with attentional evaluation of danger rather than direct motor-response inhibition (Cao & Liu, 2021; Liu et al., 2017). Accordingly, the present study interprets its behavioral findings in terms of attentional suppression and distractor processing, not as direct evidence of motor inhibitory control.

### Data analysis

Statistical analyses focused on RT and error rate. Prior to analyses, the Shapiro–Wilk test was conducted to assess the normality of the data under each condition. The results indicated that RT data in all conditions satisfied the assumption of normality (all *ps* > 0.31). Because RT analyses were restricted to Go trials, a two-way repeated-measures analysis of variance (ANOVA) was performed on mean RTs, with attentional demand (high, low) and interfering stimulus type (high-arousal dangerous, medium-arousal dangerous, low-arousal neutral) as within-subject factors.

In contrast, error-rate data deviated significantly from normality across all conditions (all *p*s < 0.01) and were therefore analyzed using nonparametric tests. Wilcoxon signed-rank tests and Friedman tests were used to examine differences in error rates as a function of interfering stimulus type, attentional demand, and response type (Go/NoGo). When a Friedman test was significant, follow-up pairwise comparisons were conducted using Wilcoxon signed-rank tests with Bonferroni correction for multiple comparisons. For clarity and consistency, errors occurring on Go trials are referred to as Go-trial errors, whereas responses made on NoGo trials (failures to withhold responding) are referred to as commission errors.

## Results

RT analyses included only correctly responded Go trials. Before the repeated-measures ANOVA, Mauchly’s test indicated that the sphericity assumption was met for interfering stimulus type [*W* = 0.995, *χ^2^* = 0.10, *df* = 2, *p* = 0.95] and for the attentional demand × interfering stimulus type [*W* = 0.89, *χ^2^* = 2.54, *df* = 2, *p* = 0.28]. Attentional demand had only two levels, so sphericity was not relevant for this factor. Accordingly, all effects were analyzed without sphericity correction. A two-way repeated-measures analysis of variance (ANOVA) on mean RTs revealed a significant main effect of attentional demand [*F*(1, 23) = 92.92, *p* < 0.001, *η*^*2*^_*p*_ = 0.80], with participants responding significantly more slowly under high attentional demand than under low attentional-demand conditions, consistent with the effectiveness of the attentional-demand manipulation. The main effect of interfering stimulus type was significant [*F*(2, 46) = 6.95, *p* = 0.002, *η*^*2*^_*p*_ = 0.23], with participants responding significantly more slowly under high-arousal dangerous conditions than under low-arousal neutral conditions (*p* = 0.003). However, the RT differences between the medium-arousal dangerous condition and low-arousal neutral (*p* = 0.62), as well as the RT differences between the medium-arousal dangerous condition and high-arousal dangerous condition (*p* = 0.08) did not reach significance. The interaction between attentional demand and interfering stimulus type was also significant [*F*(2, 46) = 3.95, *p* = 0.03, *η*^*2*^_*p*_ = 0.15].

Before the simple effects analyses, Mauchly’s tests confirmed that the sphericity assumption was met for both the high attentional-demand condition [*W* = 0.94, *χ^2^* = 1.45, *df* = 2, *p* = 0.48] and the low attentional-demand condition [*W* = 0.98, *χ^2^* = 0.42, *df* = 2, *p* = 0.81]. Sphericity corrections were therefore unnecessary for these follow-up tests. Under low attentional-demand conditions, the main effect of interfering stimulus type was not significant [*F*(2, 46) = 1.83, *p* = 0.17, *η*^*2*^_*p*_ = 0.07]. In contrast, under high attentional-demand conditions, the main effect of interfering stimulus type was significant [*F*(2, 46) = 7.81, *p* = 0.002, *η*^*2*^_*p*_ = 0.25]. Bonferroni correction *post hoc* comparisons indicated that, under high attentional demand, mean RTs in the high-arousal dangerous condition (1,801 ± 192 ms) were significantly slower than those in the low-arousal neutral condition (1,723 ± 193 ms) (*p* = 0.001), while the comparison between the medium-arousal dangerous condition (1,769 ± 200 ms) and the low-arousal neutral condition was marginally significant (*p* = 0.08). No significant difference was observed between the high-arousal and medium-arousal dangerous conditions (*p* = 0.49).

Taken together, these results indicate that the motor interference effect emerged under high attentional-demand conditions. Moreover, higher arousal levels of dangerous interfering stimuli amplified the magnitude of the motor interference effect, with the strongest interference observed for high-arousal dangerous background stimuli. Individual-level data for all participants are displayed in Figs. 2 and 3 (see Weissgerber for the rationale for displaying individual data points) (Weissgerber et al., 2015).

**Figure 2:**
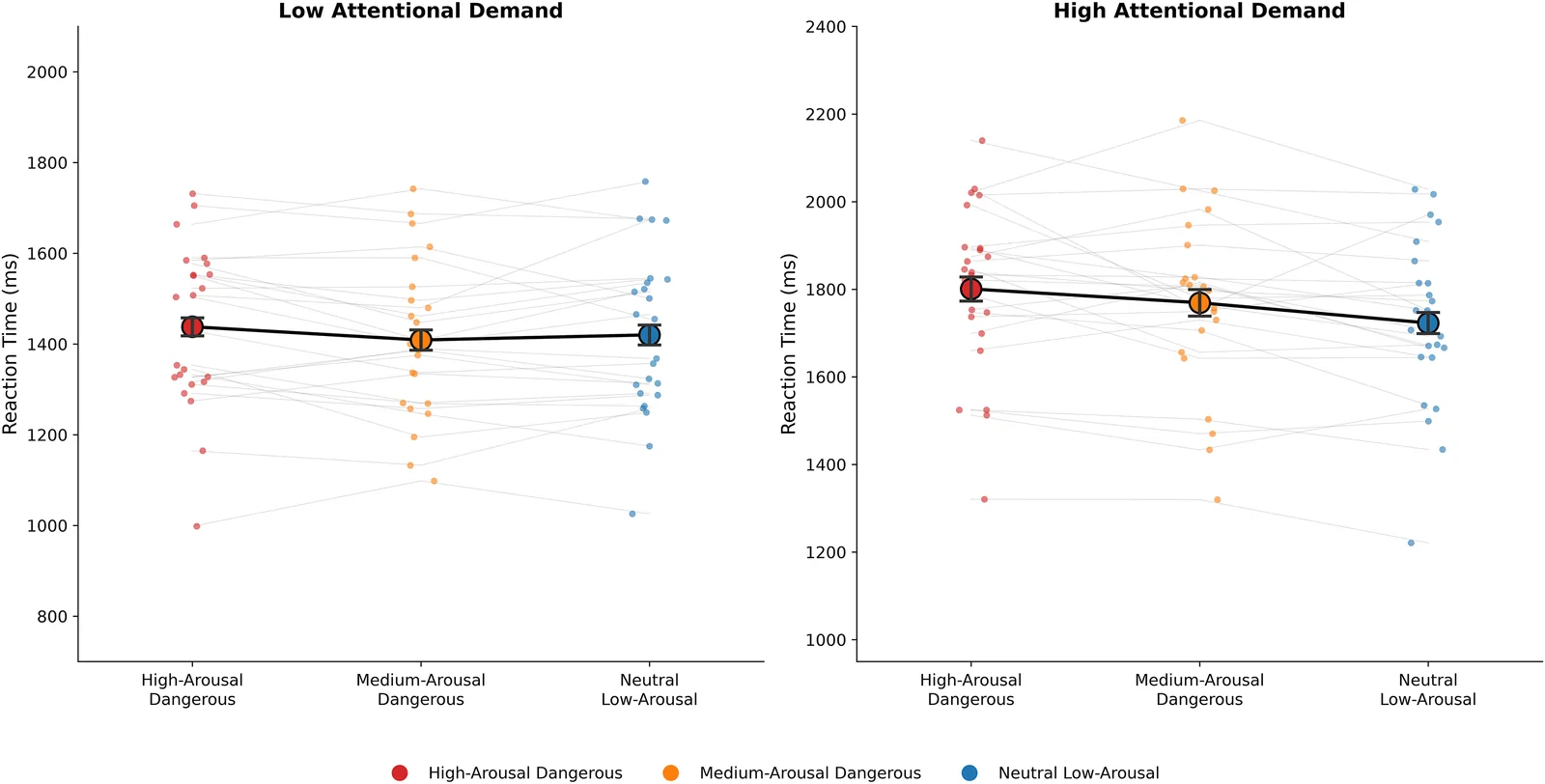
Results of the reaction times. Left panel: low attentional demand; right panel: high attentional demand. Individual dots represent each participant’s mean RT (ms) for correct Go trials, with paired observations from the same participant connected by grey lines. Large filled circles and thick lines represent group means; error bars represent within-subject 95% confidence intervals (Cousineau-Morey correction). Red = high-arousal dangerous; orange = medium-arousal dangerous; blue = neutral low-arousal. *N* = 24.

**Figure 3:**
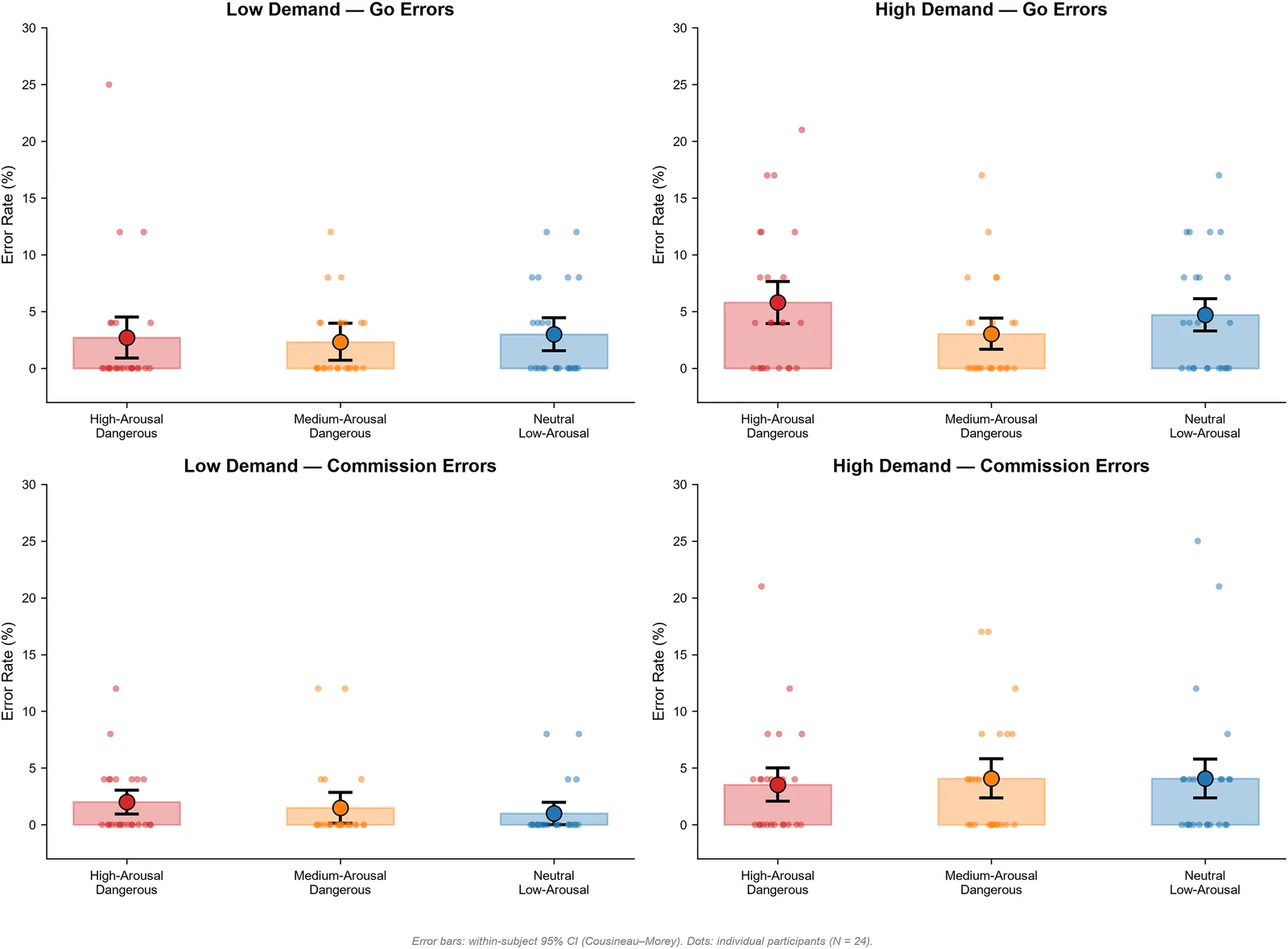
Results of the error rates. Bars represent group means; error bars represent within-subject 95% confidence intervals. Dots represent individual participants’ error rates (%). Top row: Go trial errors; bottom row: commission errors (failures to withhold on NoGo trials). Left column: low attentional demand; right column: high attentional demand. Red = high-arousal dangerous; orange = medium-arousal dangerous; blue = neutral low-arousal. *N* = 24.

In addition, nonparametric rank-based tests were conducted to examine error rates across interfering stimulus type, attentional demand, and response type (Go/NoGo) (Fig. 3). A Wilcoxon signed-rank test comparing the two levels of attentional demand revealed error rates were significantly higher under high attentional demand (*M* = 4.2%, *SD* = 4.0%) than under low attentional demand (*M* = 2.1%, *SD* = 2.4%) [*Z* = 2.69, *p* = 0.007]. These results provide further evidence for the effectiveness of the attentional-demand manipulation.

Separate Friedman tests examined Go trial error rates under each attentional-demand condition. Error rates did not differ significantly across the three interfering stimulus types under either high attentional demand [*χ^2^*(2) = 5.64, *p* = 0.06], or low attentional demand [*χ^2^*(2) = 0.72, *p* = 0.699]. For commission errors in NoGo trials, Friedman tests were not significant under high attentional demand [*χ^2^*(2) = 0.23, *p* = 0.89], or low attentional demand [*χ^2^*(2) = 3.37, *p* = 0.186]. Thus, the error-rate analyses provided no evidence that interfering stimulus type affected performance within either attentional-demand condition.

A sensitivity analysis was conducted using G*Power 3.1 (Faul et al., 2007) to determine the minimum detectable effect size for the attentional demand × interfering stimulus type interaction. With *N* = 24, *α* = 0.05, power = 0.80, an assumed correlation of *r* = 0.50 among repeated measurements, and a nonsphericity correction of *ε* = 1.0, the minimum detectable interaction effect was *f* = 0.22, corresponding to *η^2^*_*p*_ ≈ 0.05. The observed interaction effect (*η^2^*_*p*_ = 0.15) exceeded this threshold, indicating adequate sensitivity for this effect.

To mitigate order effects, the order of the high and low attentional-demand blocks was counterbalanced across participants. Block order (high-first *vs*. low-first) was included as a between-subjects factor in a 2 (attentional demand) × 3 (interfering stimulus type) × 2 (block order) mixed ANOVA. The three-way interaction was not significant [*F*(2, 44) = 0.39, *p* = 0.66, *η*^*2*^_*p*_ = 0.02], indicating that block order did not influence the results.

## Discussion

This study examined how primary attentional demand regulates the motor interference effect induced by dangerous stimuli, a question on which existing theories offer divergent predictions. The limited capacity theory holds that cognitive resources are finite and allocated according to a demand-priority principle: under low attentional demand, surplus resources remain available to process dangerous distractors, producing a motor interference effect; under high attentional demand, primary-task consumption leaves fewer resources for distractor processing, attenuating interference (Baror & Bar, 2022; Lavie & Tsal, 1994). In contrast, the signal suppression hypothesis predicts the opposite pattern: under low attentional demand, ample resources support the suppression of task-irrelevant dangerous stimuli, leading to reduced interference; under high attentional demand, suppression is compromised, allowing these stimuli to capture attention and produce greater interference.

The attentional-demand manipulation was effective, with significantly longer RTs and higher overall error rates under high than under low demand. Critically, RT interference was most pronounced under high attentional demand, where high-arousal dangerous stimuli elicited significantly slower responses than neutral stimuli (medium-arousal dangerous stimuli showed only a marginal effect, *p* = 0.08); under low demand, no RT differences emerged across stimulus types. Error rates showed no significant differences across interfering stimulus types under either demand level, although the numerical trend for high-arousal dangerous stimuli broadly paralleled the RT pattern under high demand. Thus, evidence for motor interference was confined to RTs, and the clearest contrast emerged between high-arousal dangerous and neutral stimuli under high attentional demand. However, because the high- and low-demand conditions required different target discriminations, this pattern cannot be attributed specifically to attentional demand. Differences in target-stimulus processing may also have contributed to the observed effects.

These findings converge with prior work using fearful faces and emotional scenes (Bishop et al., 2004; Bretherton et al., 2020; Erthal et al., 2006) as interfering stimuli. These studies reported that fearful-face interference was significantly stronger under high than under low attentional demand. This cross-stimulus consistency (facial expressions *vs*. threatening animals) is consistent with the view that motor interference is amplified under high attentional demand. The pattern is consistent with the signal suppression hypothesis and poses difficulties for the limited capacity theory, which would predict attenuated interference under high demand due to insufficient processing resources.

More specifically, our results are consistent with the signal suppression account: under high attentional demand, the primary task consumes a substantial share of resources (Bretherton et al., 2020), leaving insufficient capacity for top-down suppression of dangerous stimuli. Individuals are therefore unable to effectively suppress the automatic attentional capture these stimuli elicit (Sawaki & Luck, 2010). Concurrently, dangerous stimuli receive prioritized processing (Bretherton et al., 2020; Mogg et al., 2004), and their bottom-up salience dominates when suppression is compromised. The combination of reduced top-down control and enhanced bottom-up capture produces an amplified motor interference effect (Cave & Batty, 2006; LoBue & DeLoache, 2010; Theeuwes, 2010).

The findings are not easily accommodated by the core assumption of the limited capacity theory—that primary-task processing is prioritized and distractor processing depends on residual resources (Kahneman, 1973). This assumption predicts robust interference under low demand (ample residual resources) and attenuated interference under high demand (scarce residual resources). However, this prediction applies primarily to low-priority neutral distractors (*e.g*., irrelevant letters or shapes) and may not generalize to dangerous stimuli, which possess inherent processing priority (Wang & Liu, 2023; Zsido et al., 2020). Even under high attentional demand, dangerous stimuli (Cave & Batty, 2006; Fernández-Martín et al., 2017) may capture attention *via* bottom-up mechanisms (Luo, Wang & Zhou, 2022; Pessoa, Padmala & Morland, 2005), whereas neutral stimuli are more readily suppressed (Gaspelin, Leonard & Luck, 2015). These findings thus suggest a potential boundary condition of the limited capacity theory: it may account well for interference from stimuli lacking intrinsic priority but may apply less readily to biologically salient or threatening stimuli that engage prioritized processing.

The present results should also be considered in light of evidence that emotional effects on action depend on their relevance to current task goals. Previous studies found that facial expressions affected performance primarily when emotion determined the required response (Mirabella et al., 2023; Mirabella, Grassi & Bernardis, 2024; Montalti & Mirabella, 2023, 2024). This task-relevance constraint was also observed under variations in attentional demand and was synthesized in a recent review (Mirabella & Montalti, 2025). At first glance, this pattern appears to differ from the present RT results, in which interference was clearest for task-irrelevant high-arousal dangerous animal images under high attentional demand.

Several stimulus and task characteristics may contribute to this apparent difference. Previous studies by Mirabella and colleagues used facial expressions, whereas the present study used dangerous animal images. Animals such as snakes and spiders are commonly regarded as biologically salient sources of potential physical harm and may have different implications for defensive and avoidance-oriented processing (Anelli et al., 2013a; LoBue & DeLoache, 2010; Öhman, Lundqvist & Esteves, 2001). Moreover, the present paradigm spatially superimposed the background images and target display, presenting dangerous and task-relevant information simultaneously at the fovea. However, the current design cannot determine whether the different findings arose from stimulus category, spatial configuration, attentional demand, or other paradigm characteristics. We also did not manipulate task relevance or directly compare facial expressions with dangerous animal images within the same factorial design. Future studies should orthogonally manipulate task relevance, stimulus category, and attentional demand to distinguish these possibilities.

Several limitations should be acknowledged. First, the attentional-demand manipulation was confounded with target stimulus type: the high-demand condition required letter discrimination (“E” *vs*. “F”), whereas the low-demand condition required line-orientation judgment (horizontal *vs*. vertical). Although this choice produced a robust performance difference, the observed interaction cannot be attributed exclusively to attentional demand. Future studies should vary demand while holding target stimulus type constant. It remains possible that differential processing demands for letter *vs*. line-orientation discrimination, rather than attentional load *per se*, contributed to the observed effects, and an interpretation in terms of stimulus-processing differences therefore remains viable. Second, although perceived danger provides the most direct theoretical correspondence with the motor interference effect, the present stimulus categories did not orthogonally dissociate danger, IAPS normative valence, and arousal. Consequently, the observed effects cannot be attributed uniquely to perceived danger. Future studies should manipulate these dimensions independently to determine their respective contributions. Third, the equiprobable (50:50) Go/NoGo ratio limits the establishment of prepotent motor responses and constrains conclusions about motor response inhibition. The findings are therefore best interpreted in terms of attentional suppression and distractor processing. Fourth, error rates did not differ across interfering stimulus types, so evidence for motor interference was limited to RT. Fifth, the underlying neural mechanisms remain unmeasured; EEG or other physiological measures will be needed to test the proposed balance between top-down suppression and bottom-up danger processing. Finally, the final analytical sample was relatively modest (*N* = 24), and the comparison between the medium-arousal dangerous and neutral conditions under high attentional demand was marginal rather than robustly significant (*p* = 0.08). Given these considerations, the findings would benefit from replication using larger samples and designs that independently manipulate attentional demand, danger, valence, and arousal.

This study makes both theoretical and practical contributions. At the theoretical level, the findings offer preliminary observations regarding the boundary conditions of the limited capacity theory in the context of dangerous stimulus processing, suggesting that this framework may apply to low-priority neutral stimuli but may be less adequate for explaining interference from high-priority dangerous stimuli. At the applied level, the findings offer empirical guidance for cognitive resource allocation in high-risk operational settings. In low-demand scenarios (*e.g*., routine operations), ensuring adequate signal discriminability is critical to prevent missed detections. In high-demand scenarios (*e.g*., driving, precision manufacturing), balancing signal specificity against overall attentional demand may enhance hazard identification efficiency, potentially contributing to the reduction of unsafe behaviors. Future research could integrate cognitive training or intervention approaches to examine whether sensitivity to dangerous stimuli and resource-allocation efficiency under high demand can be improved through targeted training, providing a translational foundation for reducing accidents in high-risk environments.

## Conclusion

The present study suggests that attentional demand modulates the RT-based motor interference elicited by task-irrelevant dangerous stimuli. Under low attentional demand, RTs did not differ significantly across interfering-stimulus types. Under high attentional demand, RTs were significantly longer for high-arousal dangerous stimuli than for low-arousal neutral stimuli and were marginally longer for medium-arousal dangerous stimuli than for low-arousal neutral stimuli. Error rates did not differ significantly across interfering-stimulus types under either attentional-demand condition, indicating that evidence for the motor interference effect was confined to RT. This behavioral pattern is more consistent with a signal-suppression account than with the simplest prediction of limited-capacity theory, but it does not directly demonstrate attentional suppression or an inhibitory mechanism. Moreover, because attentional demand was confounded with target stimulus type, the observed interaction cannot be attributed exclusively to attentional demand. The findings should therefore be regarded as preliminary behavioral evidence and replicated using a design that manipulates attentional demand while holding target stimulus type constant.

## Additional Information and Declarations

### Data Availability

The following information was supplied regarding data availability:

The data is available at: https://doi.org/10.57760/sciencedb.36059.
